# Dipping-Press Coating Method for Retaining Transparency and Imparting Hydrophobicity Regardless of Plastic Substrate Type

**DOI:** 10.3390/polym13030403

**Published:** 2021-01-27

**Authors:** Jeeyin Park, Taekyung Lim, Keun-Hyeok Yang, Sanghyun Ju, Sang-Mi Jeong

**Affiliations:** 1 Department of Physics, Kyonggi University, Suwon 16227, Gyeonggi-do, Korea; jeeyin9404@gmail.com (J.P.); tklim@kgu.ac.kr (T.L.); shju@kgu.ac.kr (S.J.); 2 Department of Architectural Engineering Graduate School, Kyonggi University, Suwon 16227, Gyeonggi-do, Korea; yangkh@kgu.ac.kr

**Keywords:** transparent, hydrophobic, polydimethylsiloxane, plastic substrate, dipping-press coating

## Abstract

Plastics are used in cover substrates for billboards, windows, large LED signboards, lighting devices, and solar panels because they are transparent and can be colored and shaped as desired. However, when plastic cover substrates installed in outdoor environments are constantly exposed to harsh conditions such as snow, rain, dust, and wind, their transparency deteriorates owing to watermarks and dust contamination. Herein, we investigated a simple dipping-press coating method that can impart hydrophobicity while maintaining the transparency, regardless of the plastic substrate type. A highly transparent and hydrophobic coating film was formed on a plastic substrate by a two-step process, as follows: (1) application of a polydimethylsiloxane–octadecylamine coating by a dipping process, and (2) embedding (1H,1H,2H,2H-heptadecafluorodec-1-yl) phosphonic acid–aluminum oxide nanoparticles by a thermal press process. The plastic substrates on which the highly transparent and hydrophobic coating film was formed showed 150° or higher hydrophobicity and 80% or higher visible light transparency. The coating method proposed herein can easily impart hydrophobicity and is compatible with any plastic substrate that must maintain prolonged transparency without contamination when exposed to adverse conditions.

## 1. Introduction

In modern society, plastics are ideal substitutes that can augment the advantages and complement the disadvantages of existing metal and ceramic materials. Plastics, which have a lower specific gravity than metals and ceramics and high strength, are easily applicable to various designs because their transparency, coloring, and shape can be readily controlled. However, when plastics are used as covers for appliances such as billboards, windows, large LED signboards, lighting devices, and solar panels, installed in outdoor environments, dust contamination and watermarks are generated on the surface of the plastic substrate upon exposure to dust, snow, and rain, thus deteriorating the transparency of the plastic substrate. To overcome this problem, the demand for functional coating technology, which can be used to produce transparent and hydrophobic plastic substrates, is increasing. Currently, coatings with the hydrophobic polymer, polytetrafluoroethylene, are widely used, but they are expensive and poorly adhere to plastics [1,2,3]. Furthermore, according to the Cassie–Baxter model, sophisticated surface-patterning technology such as the lotus leaf structure is required to produce superhydrophobic surfaces [4,5]. Coating with metal oxide nanoparticles has disadvantages such as difficulty producing a uniform coating on a large-area substrate and the inability to maintain hydrophobicity as a result of the loss of nanoparticles upon long-term exposure to the external environment [6,7]. Furthermore, an increase in the surface roughness of the coating film to impart superhydrophobicity results in diffuse reflection of light and reduced transparency when applied to a transparent plastic substrate [8,9]. Therefore, in-depth research is needed to form highly durable hydrophobic thin films through a simple method that requires no expensive equipment and does not affect transparency.

This study introduces a smart coating method that can manifest approximately 80% or higher transparency and 150° or higher hydrophobicity through a two-step coating process (dipping and low-temperature thermocompression bonding) on transparent plastic substrates of varying thermal and mechanical properties. In general, according to the Wenzel and Cassie–Baxter models, surface energy and surface roughness are the control factors for hydrophobicity [10,11]. Hence, for the first coating, we investigated a method to form an elastic surface that has 110° or higher hydrophobicity on a plastic substrate surface using a mixed solution of polydimethylsiloxane (PDMS) and octadecylamine (ODA). For the second coating, we investigated a method to increase the surface roughness by embedding aluminum oxide (Al_2_O_3_) nanoparticles that were self-assembled with (1H,1H,2H,2H-heptadecafluorodec-1-yl) phosphonic acid (HDF-PA) on the first coating film by applying the thermal press method at 60 °C. We also investigated the durability of the hydrophobicity and transparency of the coated film on a plastic substrate upon continuous exposure to liquids. The simple and powerful coating method proposed herein should be applicable to the various commercial plastic products mentioned earlier, owing to its ability to impart stable transmittance and hydrophobicity.

## 2. Materials and Methods

### 2.1. Two-Step Coating Method Using Dipping and Thermocompression Bonding

**(*i*) First coating (PO).** After mixing 2 g of octadecylamine (ODA; technical grade, 90%, Sigma-Aldrich, St. Louis, MO, USA) and 35 g of 2-propanol (IPA; Extra Pure, 99.5%, Daejung, Siheung-si, South Korea), the mixture was heated at 90 °C for approximately 20 min in a forced convection oven (OF–02GW, Jeio Tech) until the ODA was fully melted and became transparent, to produce “solution A.” Then, 1 g of polydimethylsiloxane (PDMS) base (Sylgard 184 A, Dow Corning, Midland, MI, USA) was added to 15 g of IPA, and the solution was ultrasonicated for 15 min, following which, 0.1 g of curing agent (Sylgard 184 B, Dow Corning, Midland, MI, USA) was added to the solution to produce “solution B.” Next, PDMS–ODA (PO), the first coating solution, was prepared by mixing “solution A” and “solution B.” After dipping plastic substrates (polyethylene terephthalate (PET), polycarbonate (PC), polystyrene (PS), polymethyl methacrylate (PMMA), and polyethylene naphthalate (PEN)) with dimensions of 25 × 25 mm^2^ in a PO solution for approximately 20 s, the substrates were pulled out in a perpendicular direction to the surface of the solution and dried in a horizontal position. They were then placed in a vacuum oven (SH–VDO–08NG, SH Scientific) at 60 °C for 2 days. To remove excess PO on the film, the PO-coated substrate was immersed in IPA for approximately 10 min and then dried with an air gun. **(*ii*) Second coating (POHA).** After mixing 0.11 g of (1H,1H,2H,2H-heptadecafluorodec-1-yl) phosphonic acid (HDF-PA; 97%, SiKÉMIA, Montpellier, France) and 5 g of aluminum oxide (Al_2_O_3_; 13 nm-size nanopowder, 99.8%, Sigma-Aldrich, St. Louis, MO, USA) with 60 g of IPA, the mixture was evenly dispersed by ultrasonication for 30 min to produce an HDF-PA–Al_2_O_3_-NPs (HA) solution. The PO-coated plastic substrate was dip-coated in the second coating solution (HA solution) for approximately 20 s and dried with an air gun. Then, the substrate was inserted between two stainless-steel plates (10 mm thick) heated to 60 °C and pressed with approximately 588 N (60 kgf). The process of dip-coating and pressing the HDF-PA–Al_2_O_3_-NPs solution on the PO-coated plastic substrate was repeated twice to prepare the HDF-PA–Al_2_O_3_-NPs (HA) embedded on the PO film (POHA).

### 2.2. Optical, Wetting, and Surface Properties of a Plastic Substrate with a Highly Transparent and Hydrophobic Coating Film

The transmittance and water-contact angle (WCA) of the highly transparent and hydrophobic coating formed on a plastic substrate were measured using a UV-vis-NIR spectrophotometer (LUX-2000, Invisible Co., Suwon, Gyeonggi-do, South Korea) and a contact angle analyzer (Phoenix 300, SEO Co., Suwon, Gyeonggi-do, South Korea), respectively. Note that the volume of the water droplet was ~8 μL. The surface/cross-sectional shapes and surface roughness of the first- and second coating films were analyzed using field emission scanning electron microscopy (FE-SEM; S–4800, Hitachi, Chiyoda City, Tokyo, Japan) and atomic force microscopy (AFM; Innova, Bruker, Billerica, MA, USA). The water resistance of the highly transparent and hydrophobic coating film, resulting from continuous exposure to liquid, was examined through water spraying. For the water-spraying test, a garden hose nozzle sprayer (Nozzle 5, Takagi, Kitakyushu City, Fukuoka Pref., Japan) was placed 100 mm above the substrate, and the WCA was measured every 7.5 L while spraying a total of 120 L at a rate of 250 mL/min and speed of 170 mm/s to a PET substrate (25 × 25 mm^2^), to which a highly transparent and hydrophobic coating film was applied.

## 3. Results

**Figure 1:**
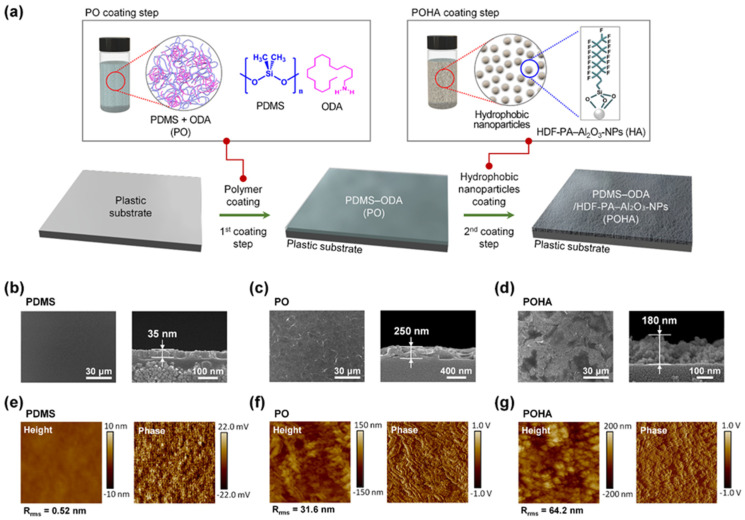
(**a**) Schematic of two-step coating method that can maintain high transparency while imparting hydropho-bicity to plastic substrates. Field-emission scanning electron microscopy (FE-SEM) images of the surfaces and cross sections of (**b**) polydimethylsiloxane (PDMS), (**c**) first coating (PO), and (**d**) second coating (POHA) formed on a polyethylene terephthalate (PET) substrate. Height- and phase-contrast atomic force microscopy (AFM) images (5 × 5 µm^2^) of (**e**) PDMS, (**f**) PO, and (**g**) POHA films formed on a PET substrate.

Figure 1a shows the schematic of a process that imparts hydrophobicity while maintaining the transparency of a hydrophilic transparent plastic substrate using the two-step coating method. For the first coating solution, a PO solution, which is a mixture of PDMS and ODA, mixed with PDMS and ODA, was used. PDMS comprises a polymer base solution and a curing agent solution. The PDMS base solution contains a poly(dimethyl-methylvinylsiloxane) prepolymer and a small amount of Pt catalyst. The curing agent solution contained poly(dimethyl-methylhydrogensiloxane) precursors and vinyl-endcapped PDMS precursors. When the PDMS base solution was mixed with the curing agent solution, the hydrosilylation reaction between the vinyl end groups of the PDMS base and the hydrosilane hydrogens of the curing agent occurred, and a 3D network was formed in the presence of the Pt catalyst [12,13]. It was recently reported that when a fabric was coated with the mixed solution of PDMS and ODA, the ODA self-assembled to form a rough wrinkled surface [14,15]. When the PDMS solution and the ODA solution are mixed, a hydrosilylation reaction between the vinyl end groups of the PDMS base and the –CH_3_ of the ODA can occur [16]. As a result, ODAs are chemically bonded with the PDMS chains, in which some crosslinked polymer network is formed. When this occurs, the hydrophilic amine groups of the ODA molecules are aggregated, and the hydrophobic PDMS chains surround them, thus generating rough and wrinkled nanostructures that can reduce the surface tension of the amine groups. Consequently, the PO films formed on the plastic substrate form a strong network due to the physical and chemical crosslinking aided by the curing agent [17].

For the second coating solution, hydrophobic HDF-PA self-assembled Al_2_O_3_-NPs solution was used. The HDF-PA self-assembled Al_2_O_3_-NPs solution was prepared by chemically bonding hydrophobic nanoparticles of 13 nm size Al_2_O_3_-NPs with HDF-PA in IPA. The thermal press method was introduced to embed the HDF-PA self-assembled Al_2_O_3_-NPs in the PO film that was first coated on a plastic substrate. After dip-coating the HDF-PA–Al_2_O_3_-NPs solution on the PO film, the substrate was inserted between two stainless steel plates heated to 60 °C and pressed at 588 N (=60 kgf). A transparent plastic substrate is a polymer that has an amorphous structure. Even with the same chemical structure, transparent amorphous polymers have lower thermal resistance than opaque crystalline polymers. In this study, a stainless-steel plate heated to 60 °C made the PDMS matrix of the PO film, formed by the rubberier first coating, while maintaining transparency without heat deflection of the transparent plastic substrate (PET, PC, PS, PMMA, and PEN). Therefore, during the process of pressing the POHA film, the HDF-PA–Al_2_O_3_-NPs (HA) coated on the PO film were embedded more deeply into the rubbery PO film.

Figure 1b–d shows the FE-SEM images of PDMS, PO (first coating), and POHA (second coating), respectively, coated on a commercial transparent PET substrate. When only PDMS was coated on PET, a uniform ~35-nm-thick coating film was formed, and the surface was very flat. Meanwhile, the PO-coated PET surface was rough because the nanostructures formed protrusions, which appeared owing to the phase separation of the amine groups of ODA in the PDMS matrix. The thickness of the PO film dramatically increased to ~250 nm compared that of the PDMS film (~35 nm) because the PO film was coated with the ODA-assisted crosslinked polymer, whose molecular weight and viscosity were higher than those of PDMS. The POHA films coated on the PET substrate showed increased roughness compared to the PO film because of the randomly aggregated Al_2_O_3_-NPs. The FE-SEM image of its cross section confirmed the increased surface roughness and the presence of the HDF-PA–Al_2_O_3_-NPs embedded inside the PO film. Note that some HDF-PA–Al_2_O_3_-NPs were exposed on the surface of the PO film. The thickness of the POHA film was ~180 nm was lower than the thickness of the PO film (~250 nm). This can be attribute to the pressing force of 588 N (60 kgf) applied to the PO film when it was embedded with the HDF-PA–Al_2_O_3_-NPs.

Figure 1e–g shows the surface morphologies of the PDMS, PO, and POHA films, respectively, coated on the PET substrate. The height-contrast AFM images (size of 5 × 5 µm^2^) showed that the surface of the PDMS became very flat with a root-mean-square roughness (*R*_rms_) value of 0.52 nm. In addition, the surfaces of the PO and POHA films rapidly became rougher with *R*_rms_ values of 31.6 and 64.2 nm, respectively. The surface roughness of PO films was caused by the aggregation and physical crosslinking by ODA that additionally occurs during the crosslinking process of PDMS. Consequently, the aggregations of the ODA molecules anchored on the PDMS polymer chains generated stress inside the PDMS matrix, causing the surface of the PO film to wrinkle. The roughness of the POHA appeared through the grain-shaped, aggregated HDF-PA–Al_2_O_3_-NPs embedded on the rough and rubbery PO film. The roughness and surface energy of granular-aggregated HDF-PA–Al_2_O_3_-NPs can make the surface of POHA film sufficiently hydrophobic. Nevertheless, the reason for using a rough and rubbery PO film is to enhance the durability of the hydrophobic POHA film by increasing the adhesion and contact area between PO and HA.

In general, during AFM measurements, the coulombic and van der Waals interactions between the microscope tip and the film surface change the movement of the cantilever/tip assembly, and the position change in the laser, reflected from the back of the cantilever, is imaged. At this time, phase-contrast images are obtained by detecting the phase shift of the oscillation signals that were changed after the interaction between the free oscillation signal of the cantilever and the film surface. The phase-contrast image is changed by the chemical/physical properties, such as composition, adhesion, and viscoelasticity of the sample surface [18]. In Figure 1e–g, the phase-contrast images show the boundary of the zigzag wrinkles more clearly in the height-contrast image. This demonstrates that the roughness of the PO and POHA films increased. The entire area of the phase-contrast image of the PO film appeared in similar colors, which suggests that only the same material is exposed to the surface. This means that phase-separated ODA exists inside the PDMS matrix without being exposed to the surface.

**Figure 2:**
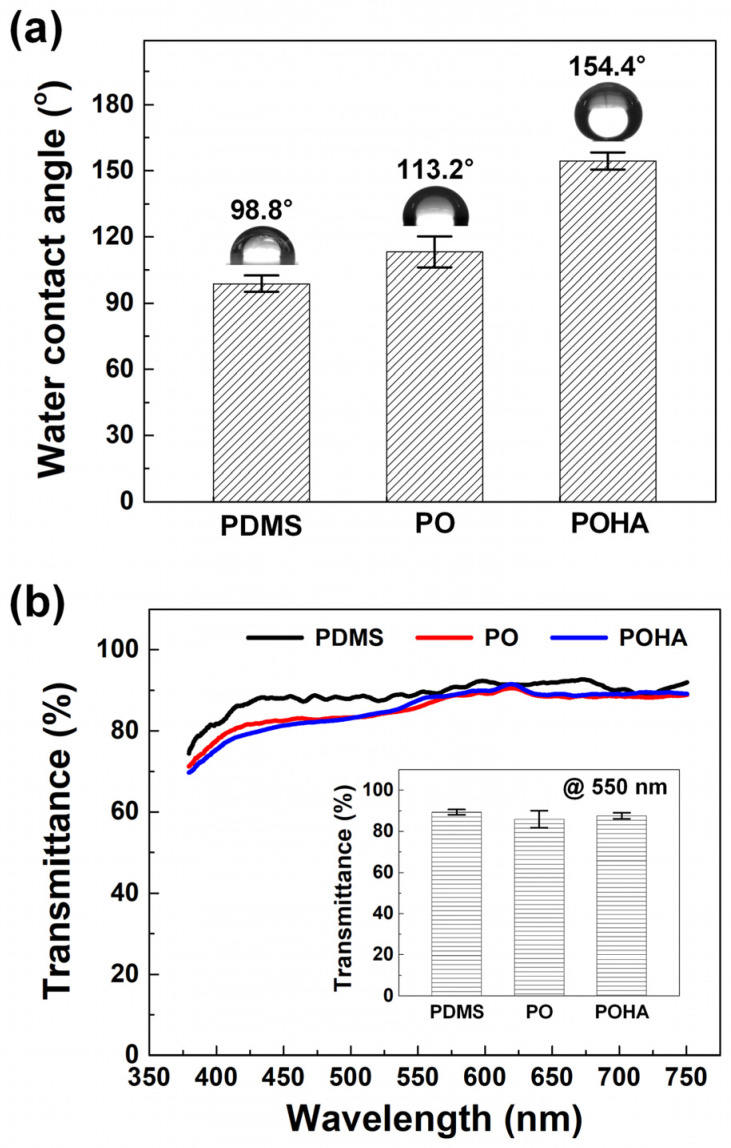
(**a**) Water-contact angles (WCAs) and (**b**) transmittance of PET substrates coated with PDMS, PO, and POHA films.

To observe the changes in hydrophobicity and transparency of the case where only the first coating was performed, and the case where the first and second coatings were performed on transparent plastic substrates, PET substrates were coated with PDMS, PO, and POHA. The WCAs of the PDMS, PO, and POHA-coated PET substrates were 98.8 ± 3.7°, 113.2 ± 7.0°, and 154.4 ± 3.9°, respectively. (Figure 2a) Thus, the hydrophobicity increased in proportion to the roughness (0.52, 31.6, and 64.2 nm, respectively). The hydrophobicity values varied according to the Cassie–Baxter models because of the different surface energy and roughness values of the PO and POHA films [10,11]. Maintaining the transparency of the substrate even after applying the hydrophobic coating film is very important in terms of commercialization. Figure 2b shows the transmittance of PDMS, PO, and POHA-coated PET substrates. At a wavelength of 550 nm, the transparency values of the PDMS, PO, and POHA films coated on the PET substrate were 89.3 ± 1.3%, 85.9 ± 4.2%, and 87.5 ± 1.5%, respectively. The transmittance of POHA was slightly higher than that of PO, because the film thickness (180 nm) of the former was smaller than that of the latter (250 nm). This is because, as the thickness of the thin film increases, the optical transmittance decreases, and the possibility of forming a structurally heterogeneous thin film increases [19]. Although the POHA film is the addition of HDF-PA–Al_2_O_3_ NPs to the PO film, the Al_2_O_3_ NPs with a size of ~13 nm show very little scattering in the visible region. This is because the scattering of light for the wavelength in the visible region is very small in NPs with a diameter of ~10 nm; scattering occurs most in the vicinity of 300 nm diameter, and the scattering decreases as the NPs become larger [20]. This confirmed that the application of the POHA film could provide the substrate with hydrophobicity of 150° or higher, and a transmittance of 80% or higher.

**Figure 3:**
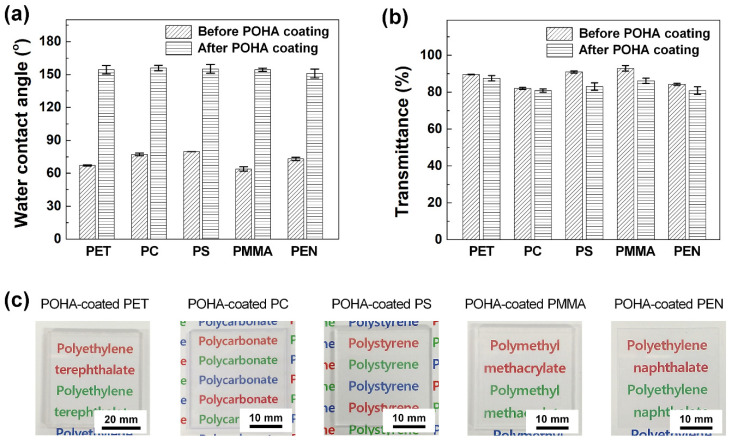
(**a**) WCAs and (**b**) transmittance of plastic substrates (PET, PC, PS, PMMA, and PEN) before/after POHA coating. (**c**) Photographs of five POHA-coated plastic substrates (PET, PC, PS, PMMA, and PEN). The colored letters printed on the paper under the plastic substrates are clearly visible.

To verify the applicability of the POHA film regarding its hydrophobicity and high transparency in various transparent plastic substrates, it was applied to five samples (PET, PC, PS, PMMA, and PEN). Figure 3a shows that the WCAs of the transparent plastic substrates before/after POHA coating were 67.1 ± 0.6°/154.4 ± 3.9° (PET), 77.2 ± 1.3°/156.0 ± 2.5° (PC), 79.7 ± 0.01°/155.2 ± 4.1° (PS), 63.9 ± 2.0°/154.4 ± 1.5° (PMMA), and 73.1 ± 1.5°/151.0 ± 4.0° (PEN). All substrates showed different WCAs owing to their varying roughness before they were coated with POHA, but a coating of POHA yielded uniform WCAs in the range of 151° (min)–156° (max). Figure 3b shows the transmittance values before and after applying the POHA coating to the five transparent plastic substrates (PET, PC, PS, PMMA, and PEN). Their transmittance values before and after POHA coating were 89.6 ± 0.1%/87.5 ± 1.5% (PET), 82.0 ± 0.5%/80.8 ± 0.9% (PC), 90.9 ± 0.6%/83.0 ± 2.0% (PS), 92.9 ± 1.5%/86.1 ± 1.4% (PMMA), and 84.3 ± 0.6%/80.9 ± 2.1% (PEN). The thermal press process was performed at 60 °C, which was higher than the glass transition temperature (*T*_g_) value of the PDMS (approximately −120 °C), and lower than the *T*_g_ values of the plastic substrates (*T*_g_ values of PET, PC, PS, PMMA, and PEN are approximately 76, 147, 100, 105, and 120 °C, respectively) [21,22,23,24]. Thus, the transparency of the substrate itself could be maintained because heat deflection did not occur on the lower plastic substrate. However, the transmittance decreased after coating with POHA, with the difference ranging from 1.2% (min) to 7.9% (max). This can be attributed to the uneven thickness of the POHA film, as a result of the manually performed dipping-press method. It is notable that our proposed coating method is simple, and does not require expensive equipment or trained professionals. Although there are slight reductions in the transmittance values due to coating being performed manually, the values are within an acceptable margin of error. Figure 3c shows photographs of the PET, PC, PS, PMMA, and PEN substrates coated with POHA. The colored letters printed on the paper below the plastic substrates can clearly be seen. In general, imparting 150° or higher hydrophobicity to a solid surface requires the formation of surface roughness with micro/nano-scale structures and chemically low surface energy. If micro/nanoscale structures are formed to increase the surface roughness, the transmittance of the solid surface is decreased greatly, owing to the blocking, scattering, and interference of light by micro-/nano-scale structures. However, the two-step coating method used in this study could manifest hydrophobicity, while imparting low surface energy and surface roughness using PDMS–ODA and HDF-PA–Al_2_O_3_-NPs, and prevent significant changes in the transparency reduction rate of the substrate itself.

**Figure 4:**
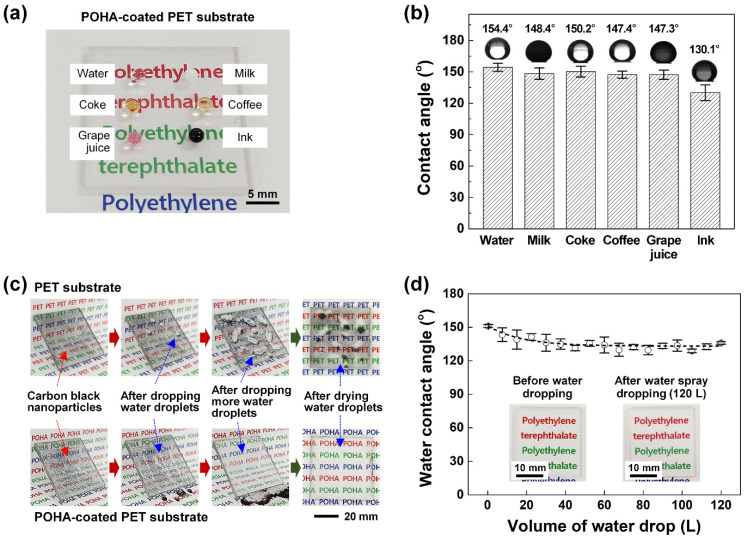
(**a**) Photographs showing that water, milk, Coke, coffee, grape juice, and ink solutions form droplets without spreading on the POHA-coated PET substrate. (**b**) WCAs of the water, milk, Coke, coffee, grape juice, and ink solutions on the POHA-coated PET substrate. (**c**) General PET substrate showing carbon black nanoparticle marks left by water and the POHA-coated PET substrate showing carbon black nanoparticle cleaned by water. (**d**) Change trend of WCAs of the POHA-coated PET substrate according to continuous water drops. The inset shows photo images of the POHA-coated PET substrate before and after the water dropping test.

Figure 4a shows a photograph of the formation of droplets of six solutions that are encountered in everyday life (water, milk, Coke, coffee, grape juice, and ink) on a POHA-coated PET substrate. Owing to the hydrophobicity of the POHA-coated substrate, the droplets of the solutions could maintain round shapes without spreading. Furthermore, the color text “polyethylene terephthalate” printed on the paper below the POHA-coated PET substrate could be observed clearly without optical distortion. This suggests that POHA did not have a significant effect on transparency. The measured contact angles of the POHA-coated PET substrate were 154.4 ± 3.9°, 148.4 ± 5.3°, 150.2 ± 5.0°, 147.4 ± 3.4°, 147.3 ± 4.4°, and 130.1 ± 7.4° for water, milk, Coke, coffee, grape juice, and ink, respectively (Figure 4b). This confirmed that the POHA film had excellent wettability, not only for water, but also for various solutions used in everyday life.

In general, surfaces of lighting covers, solar panels, and architectural glasses installed outdoors are easily contaminated due to long-term exposure to external environments. Particularly, when moisture is added by snow or rain, dust stains the substrate, not only damaging the aesthetic, but also greatly reducing the efficiency of lighting and solar power generation. Contamination due to dust or foreign substances directly affects solar power generation, reducing as much as approximately 10% of the power generation. Note that dust reflects or absorbs solar radiation. If the POHA coating proposed in this study is applied to external transparent substrates, it can significantly reduce the attachment rate of dust or foreign substances because it can impart hydrophobicity to the surface without significantly reducing transparency. When the surface of the general transparent PET substrate (Figure 4c) was smeared with carbon black nanoparticles and washed with water, stains remained owing to the particles corresponding to the dust. In contrast, no stains remained on the POHA-coated transparent PET substrate because the particles were washed away with water.

One of the most significant disadvantages of hydrophobic surface coating using general chemical treatments or metal oxide nanoparticles is poor durability, wherein hydrophobicity is severely degraded upon continuous exposure to external conditions, such as snow or rain. This is because the hydrophobic film or a metal oxide nanoparticle is weakly bonded with the surface of the lower substrate and is vulnerable to peeling and abrasion [25,26,27]. This study improved the bonding force between the PO film and the lower substrate through the thermal press process, which embedded the hydrophobic HDF-PA–Al_2_O_3_-NPs inside the rubbery PO film. This prevented the peeling of the hydrophobic HDF-PA–Al_2_O_3_-NPs and PO film from the lower substrate, even in an environment that was continuously exposed to water. To verify if the POHA-coated PET substrate retained its hydrophobicity in continuous contact with water, the WCA was measured every 7.5 L, while 120 L of water was sprayed at a rate of 250 mL/min onto the substrate (25 × 25 mm^2^) from a height of 100 mm. As seen in the wetting property shown in Figure 4d, the WCA value decreased slightly from 151.2° ± 1.3° at first to 143.4° ± 6.2° (7.5 L), 139.1° ± 8.6° (15 L), and 136.2° ± 7.6° (30 L), but after 37.5 L to 120 L, the WCA value remained constant at 133.0° ± 2.4°. The phosphonates of the HDF-PA self-assembled monolayers with phosphonic acid anchor groups formed metal–ligand coordination bonds with Al_2_O_3_ and induced strong chemical and thermal bonds. However, the water spray washed away the HDF-PAs that did not form strong bonds with Al_2_O_3_; as a result, the WCA decreased by ~10% compared to the beginning [28]. As shown in the inset in Figure 4d, the transmittance of the POHA-coated PET substrate did not change significantly after the water spray test.

## 4. Conclusions

In this study, we proposed a simple coating method that can increase hydrophobicity, while maintaining the transparency of plastics. In step 1, the PO film with a thickness of several hundred nanometers, in which PDMS and ODA were mixed, was formed on the plastic substrate through the dipping process. In step 2, HDF-PA self-assembled Al_2_O_3_-NPs were embedded in the PO film using the thermal press method at 60 °C, which did not cause heat deflection to the plastic substrate and PO film. This two-step coating process of the dipping-press could form a hydrophobic transparent coating on the plastic substrate. The POHA film could be applied to various transparent plastic substrates, such as PET, PC, PS, PMMA, and PEN and showed a 150° or higher hydrophobicity and 80% or higher transparency. Furthermore, the formed hydrophobic transparent coating film showed contact angles greater than 130° for various solutions used in everyday life such as milk, Coke, coffee, grape juice, ink, and water. The simple coating method proposed in this study is expected to provide a good alternative to transparent thin film coatings that can not only solve the aesthetic problem of indoor/outdoor plastic substrates that are vulnerable to contamination, but retain the transparency and efficiency of the commercial plastic products as well.

## Data Availability

The data presented in this study are openly available.
